# Data on the impact of an object with different thicknesses of different soft materials at different impact velocities on a dummy head

**DOI:** 10.1016/j.dib.2019.103885

**Published:** 2019-04-01

**Authors:** Ahmad Yaser Alhaddad, John-John Cabibihan, Ahmad Hayek, Andrea Bonarini

**Affiliations:** a Qatar University, Department of Mechanical and Industrial Engineering, Doha, 2713, Qatar; b Politecnico di Milano, Department of Electronics, Information and Bioengineering, Piazza Leonardo da Vinci 32, Milano, 20133, Italy

**Keywords:** Soft materials, Severity indices, Head impact, Safety

## Abstract

The purpose of this data is to investigate the effect of different thicknesses of different soft materials samples added to an object on the resultant head acceleration of a developed dummy head upon impact. The object was a cylinder (10 × 10 cm^2^, diameter and height) and weighs 0.4 kg. The investigated materials were Ecoflex, Dragon Skin, and Clay while the thickness were 1 mm, 2 mm, 3 mm, and 5 mm. The velocities of the impacts for the 108 experiments were between 1 m/s and 3 m/s. Three severity indices (i.e. peak head linear acceleration, 3 ms criterion and the Head Injury Criterion (HIC)) were calculated from the raw acceleration data. The impact velocities were tabulated from the video recordings. A summary of the processed data and the raw data are included in this dataset. Online repository contains the files: https://doi.org/10.7910/DVN/TXOPUH.

**Table:** **Specifications Table** [Please fill in right-hand column of the table below.]

Subject area	Engineering
More specific subject area	Safety
Type of data	Dataset and tables
How data was acquired	From a low-cost developed experimental setup that contains a 3D-printed dummy head. The head contains a tri-axial accelerometer that was used to acquire the changes in the accelerations. The accelerometer was connected to a computer through a data acquisition card.
Data format	Raw and processed.
Experimental factors	Three factors were considered: the material type, its thickness, and the impact velocity.
Experimental features	The dummy head's accelerations were generated upon impact, stored, and then analyzed. The impact velocities were calculated based on the video recording of each experiment.
Data source location	Qatar University, Doha 2713, Qatar.
Data accessibility	https://doi.org/10.7910/DVN/TXOPUH.
Related research article	A.Y. Alhaddad, J.J. Cabibihan, A. Bonarini, Head impact severity measures for small social robots thrown during meltdown in autism. Int. J. Soc. Robot. (2018) 1–16. https://doi.org/10.1007/s12369-018-0494-3[2], Ahmad Yaser Alhaddad, John-John Cabibihan, Ahmad Hayek, Andrea Bonarini, "Safety experiments for small robots investigating the potential of soft materials in mitigating the harm to the head due to impacts," SN applied Sciences, Springer Nature, 2019, 1: 476, https://doi.org/10.1007/s42452-019-0467-7[7]

**Table:** 

**Value of the Data** • The data could be used generally for safety purposes to understand head's acceleration due to impacts. • The data could be used to investigate the potential of soft materials in reducing any harm to the head. • Optimizing toys' designs to improve their safety.

## 1 Data

The Harvard Dataverse link contains a readme file, raw data files, and a summary file [1]. The readme file provides a detailed description about the raw data files and the summary file. The raw data files composed of the National Instruments (NI) TDMS files that contain the raw acceleration readings reported in the gravitational acceleration (g = 9.81 m/$s^{2}$). The summary file contains the processed raw acceleration readings categorized according to the materials investigated and their thicknesses. Furthermore, it contains the analysis for the three severity indices.

The file ***LabView_raw_acceleration_data.rar*** in the online repository contains the raw acceleration readings of the embedded sensor (i.e. accelerometer). This file contains three subfolders, namely clay, dragon skin, and ecoflex. Each material folder is further subdivided to four subfolders based the thicknesses considered, which were 1 mm, 2 mm, 3 mm, and 5 mm. Finally, each folder contains 9 TDMS^1^ files representing 9 different experiments.

The columns inside the TDMS files and their corresponding descriptions are summarized in Table 1.

**Table 1:** Description of the raw data columns in the TDMS files.

Column	Name	Description
C	Voltage_2 (Formula Result)	The resultant linear acceleration of the dummy head (Unit is in g)
D	Voltage_1 (Formula Result)	The force sensor readings (Unit is in N) **Note:** Not used.
E	Voltage_2 (Formula Result)1	The X acceleration (Unit is in g)
F	Formula Result	The Y acceleration (Unit is in g)
G	Formula Result 1	The Z acceleration (Unit is in g)

^**1**^**Note**: Add-In from National Instruments is needed to view the TDMS files in Excel: http://www.ni.com/example/27944/en/.

The file ***processed_summary.xlsx*** contains a summary of the processed data categorized by tabs based on the material tested (i.e. ecoflex, dragon skin, and clay). Each tab contains the thicknesses considered (i.e. 1 mm, 2 mm, 3 mm, and 5 mm) and the corresponding analysis for the peak head linear acceleration (g), the 3 ms criterion (g), and the Head Injury Criterion (HIC). Additionally, it contains the analysis for the impact velocity (m/$s^{2}$) for each experiment.

## 2 Experimental design, materials, and methods

### 2.1 Experimental setup

**Fig. 1:**
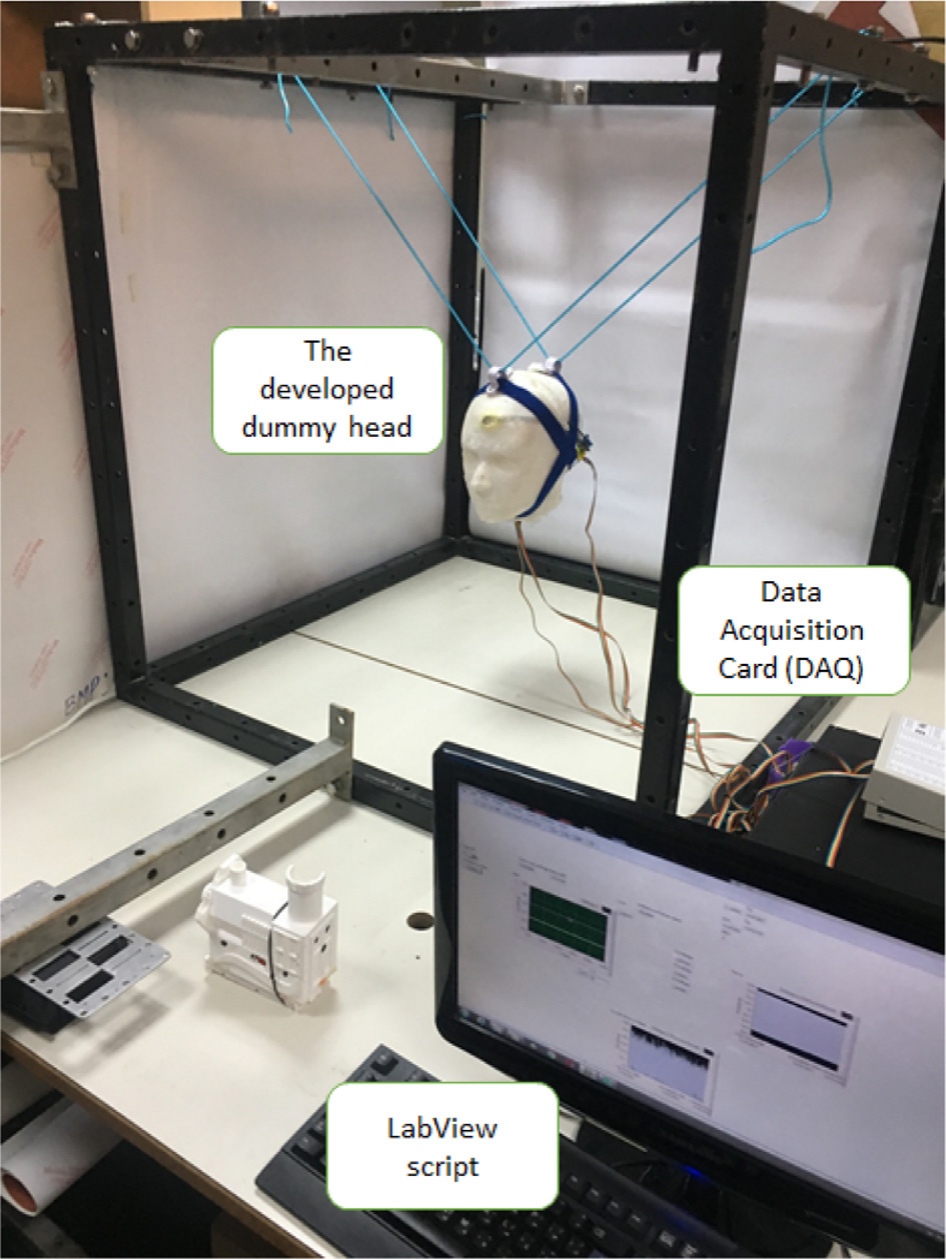
The experimental setup [2].

The experimental setup was based on a low-cost developed dummy head. The dummy head was 3D-printed that was made of polylactide (PLA). Clay was added to make the dummy head reach a mass of 3.1 kg, which is very close to that of children's dummy heads. The accelerations of the head were measured with a tri-axial accelerometer embedded inside the head. This head model was placed in a dedicated frame with ropes (Fig. 1). The readings of the sensor were read at 20 kHz through a data acquisition card. Our earlier studies provided more detailed overview of the experimental setup [2], [3].

### 2.2 Impactor preparation

**Fig. 2:**
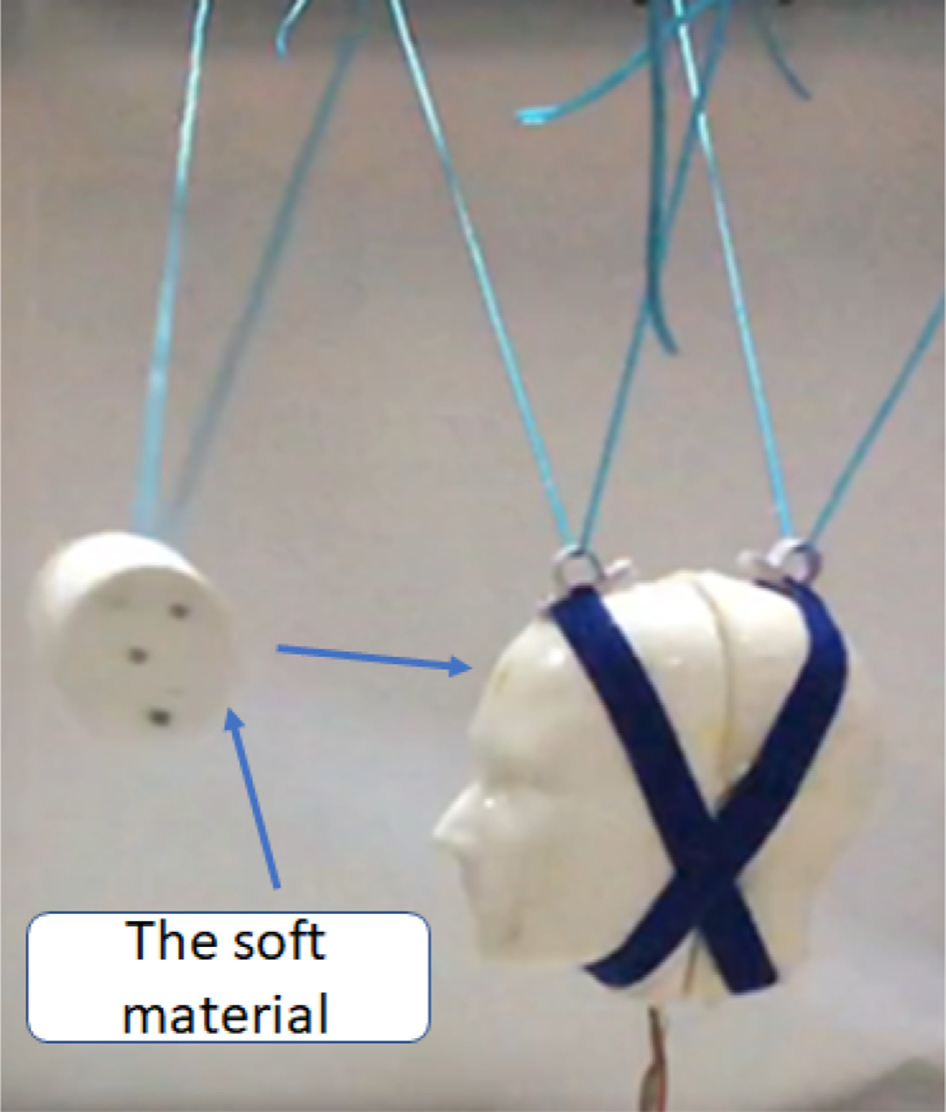
A sample of the experiments [7].

The impactor used was cylindrical in shape (10 × 10 $cm^{2}$) and weighs 0.4 kg (Fig. 2). Dimensions selected are within the expected dimensions of that of small robots or toys [4], [5], [6]. The impactor was built using a 3D printer. Samples (5 × 8 $cm^{2}$) of the soft materials (i.e. Clay, Ecoflex OO-30, and Dragon skin FX Pro) were attached to the area of impact (Fig. 2).

### 2.3 Procedures

A total of 108 impact experiments were conducted covering all the three soft materials and the four thicknesses. Tying the object to the frame allowed the generation of different impact velocities by changing the height at which the object is dropped. This approach achieved consistency in terms of the impact velocities levels across all experiments. All impacts were recorded in slow-motion (240 fps, 720 pixels) and an open-source video software (Tracker version 4.10.0) was used in the analysis for impact velocities. A LabView script was used in acquiring and storing the raw data while a MATLAB script was used to post-process it for the three severity indices.
